# Determinants of Sick and Dead Pig Waste Recycling—A Case Study of Hebei, Shandong, and Henan Provinces in China

**DOI:** 10.3390/ani12060775

**Published:** 2022-03-19

**Authors:** Xu Ge, Apurbo Sarkar, Si Ruishi, Md Ashfikur Rahman, Jony Abdul Azim, Shuxia Zhang, Lu Qian

**Affiliations:** 1 College of Economics and Management, Northwest A&F University, Yangling 712100, China; xuge@nwafu.edu.cn (X.G.); apurbo@nwafu.edu.cn (A.S.); 2 School of Public Administration, Xi’an University of Architecture and Technology, Xi’an 710018, China; siruishi@126.com; 3 Development Studies Discipline, Social Science School, Khulna University, Khulna 751013, Bangladesh; ashfikur@ku.ac.bd; 4 School of International Education, Xidian University, Xi’an 710071, China; abdulazim.jony@yahoo.com; 5 College of Veterinary Medicine, Northwest A&F University, Yangling 712100, China

**Keywords:** personal commitment, reward and punishment mechanism, farmers, waste recycling behavior

## Abstract

**Simple Summary:**

Proper handling of dead and sick pig carcasses is a significant concern for farmers, the general public, academia, and government. By drawing on the existing literature, the study selects various determinants of sick and dead pig recycling and evaluates the impacts of rewards and punishment mechanisms on farmers’ commitment to proper handling of dead and sick pigs. We utilized face-to-face discussion accompanied by a structured questionnaire to grasp the opinion of the Chinese pig farmers. The article elucidates the moderating effects of reward and punishment mechanisms, which will be crucial for understanding the on-hand experience of the farmers towards dead and sick pig recycling.

**Abstract:**

Improper handling of sick and dead pigs may seriously affect public health, socio-economic conditions, and eventually cause environmental pollution. However, effective promotion of sick and dead pig (SDP) waste recycling has become the prime focus of current rural governance. Therefore, the study explores the impact of commitment, rewards, and punishments to capture the recycling behavior of farmers’ sick and dead pig waste management. The study employs factor analysis, the probit model, and the moderating effect model to craft the findings. The study’s empirical setup comprises the survey data collected from the Hebei, Shandong, and Henan provinces, representing the major pig-producing provinces in China. The study found that the commitment, reward, and punishment mechanisms are essential factors affecting the farmers’ decision-making on recycling sick and dead pig waste. The marginal effect analysis found that the reward and punishment mechanism is more effective than the farmers’ commitment. The study confirmed that in the recycling treatment of sick and dead pig waste, the farmers’ commitment and the government’s reward and punishment policy are the main factors that influence farmers to manage sick and dead pig waste properly. Therefore, the government should highlight the importance of effective waste management, and training facilities should also be extended firmly. The government should impose strict rules and regulations to restrict the irresponsible dumping of farm waste. Monitoring mechanisms should be put in place promptly.

## 1. Introduction

The resource utilization of agricultural waste is an integral part of rural environmental governance [1]. In China, it is estimated that about 60 million pigs die from diseases such as African swine fever and anthrax each year [2], and the proportion of concentrated professional and harmless treatment is also relatively lower [3,4]. The frequent occurrence of such diseases eventually becomes a major concern for the government, epidemiologists, academia, and the public. This agricultural waste that has not been recycled and treated harmlessly is massive, usually piled up and burned randomly, eventually impacting the urban and rural environment severely [5]. The waste recycling treatment of sick and dead pigs is one of the critical links in the clean production of pig breeding [6]. Especially when encountering significant diseases such as African swine fever, the mortality rate will increase and bring many pig carcasses. If such sick and dead pig products are not treated harmlessly and adequately, it will seriously affect the quality and safety of pork and may cause further spread of the epidemic, threatening public health and pig production safety [7]. It will also cause ecological and environmental problems, which predominantly result in severe pollution to the soil, water, and air [8]. However, how to deal with sick and dead pigs rationally has become a focal point within the environmental management of livestock and poultry breeding [9].

At present, there are two treatment methods for sick and dead pigs which are popular among farmers: one is simple treatment represented by deep burial and incineration, and the other is waste recycling implemented using technologies such as carbonization, chemical production, composting, and fermentation [10,11]. However, simple treatment can cause secondary soil, water, and air pollution, but scientific waste recycling can avoid these forms of pollution [12]. It is a scientific path to realize resource recycling, ecological protection, and green, healthy and sustainable development [13,14]. However, in China, the degree of recycling of sick and dead pig waste is still deficient [15]. The farmer is in charge of deciding whether to adopt waste recycling treatment for sick and dead pigs or not. Therefore, from the farmer’s perspective, identifying the influencing factors of the treatment of sick and dead pig wastes has become one of the critical issues among the government and academia [10]. Interestingly, there are very few studies evaluating the influencing factors on the recycling and effective waste disposal behavior of sick and dead livestock and poultry carcasses [16,17]. However, recycling decisions concerning sick and dead livestock and poultry carcasses may act as a kind of pro-environmental behavior that can generate positive environmental externalities.

The conflicts brought by the externalities cause farmers to face anarchy or stimulus market conditions, as greater economic interests are involved, and they tend to choose non-standard treatment instead of harmless treatment [18,19]. As most of the farmers tend to seek cost-effectiveness in their core farming tactics, they will eventually avoid more environmentally friendly but more costly waste recycling treatment methods [20]. Therefore, in the absence of external economic stimuli, governmental supervision, support, and incentives are required [21]. However, because farmers who directly engage in livestock and poultry breeding have a relatively lower awareness of the relevant government policies and regulations, the implementation effects have been weakened to a certain extent [22,23]. In China, there are substantial deviations and shortcomings in the existing regulatory policies and subsidy mechanisms [24,25]. For example, every province has its unique structure and political agenda. Some provinces help farmers with monetary subsidies, whereas others provide credit and resource subsidies. Moreover, there is a huge gap between the reward and punishment strategies as well. The reward and punishment mechanisms may play a crucial role in maximizing the adoption of harmless management of sick and dead pig waste and promoting resource utilization [26,27,28].

In pig breeding, farmers have to face the situation where they need to handle sick and dead animals, as it is an unavoidable biological process [29]. However, the waste including dead and sick pigs must be managed scientifically to reduce potential microbial threats and eventually ensure environmental protection and public health [30]. In this process, farmers should be careful to ensure that the selected treatment method can prevent disease transmission and environmental pollution [31,32]. Therefore, the notion brings an interesting concept of harmless treatment [11]. Harmless treatment refers to the process of using combined physical and chemical-based treatment methods that can eliminate pathogens and are also safe for the environment, humans, and other animals [33]. However, the dead pig carcasses may be considered farm waste and potential biomass resources. If those resources can be handled effectively and adequately, farmers can avoid several issues such as environmental damage and maintain proper food, health, and public safety precautions. However, simple treatment can effectively dispose of corpses, but it cannot utilize the resources effectively [33]. Traditional methods, such as deep burial and corpse wells (pools), occupy land resources, causing severe air, soil, and water source dgeadation and hindering biological safety [34]. Although the standard incineration method can kill pathogenic microorganisms, it consumes a large amount of energy and produces harmful substances such as nitrogen oxides, sulfides, and dioxins, which can easily cause secondary pollution [35]. Thus, the concept of waste recycling can be considered as one of the potential methods to handle pathogenic microorganisms, treat corpses, and turn waste into resources [36,37].

The Chinese government has taken various policies and measures that pay equal attention to rewards and punishments for promoting the recycling of sick and dead pig waste [38]. On the one hand, the government has imposed relatively strict laws and regulations prohibiting the non-standard handling of sick and dead pigs [39]. The government circulated the “Technical Specification for the Harmless Treatment of Diseased and Dead Animals” to build farmers’ awareness levels. The circulation would be helpful for the authorities to ensure that a farmer who violates laws and regulations for managing sick and dead pigs will face punishment [39]. On the other hand, the Chinese government also provides farmers with subsidies to encourage them to choose innovative, high-efficiency, green, and environmentally friendly harmless treatment methods [40]. In China, harmless treatment methods of sick and dead pigs are dominated by several simple methods such as deep burial and incineration [41]. However, various internal and external issues such as improper treatment and disposal, inadequate technology, imperfect compensation mechanisms, and lack of supervision continuously and negatively affect the effectiveness of the methods mentioned above [42]. Interestingly, with gradual standardization, widespread concerns of environmental protection, and resource utilization potentiality, harmless treatments might effectively manage the dead and sick pig waste effectively [43].

The existing literature mainly focuses on the effect of reward and punishment mechanisms and the impact of commitments on farmers’ pro-environmental behaviors in an isolated manner [44,45,46]. However, farmers’ commitments, reward and punishment mechanisms, and farmers’ waste recycling behaviors have not been explored yet under the same framework. The study will be the first attempt to cover this crucial research gap to the best of our knowledge. The farmer’s commitment, reward, and punishment mechanisms may not be independent when influencing the behavior of farmers. Moreover, the farmer’s commitment is more effective under the reward and punishment mechanism [47]. The uneven distribution and low-level awareness of farmers’ commitments are still tricky problems in the current academic research. The study intends to explore the following research questions: (i) how effective are the commitment and reward and punishment mechanisms? (ii) How does the farmer’s commitment guarantee its effectiveness under the influence of the reward and punishment mechanism? (iii) What is its specific impact mechanism? The study explored the relationship between farmers’ commitments, rewards and punishments, and the recycling behavior of sick and dead pig waste. The main innovative contributions of the study are (i) to calculate the extent to which the government’s reward and punishment policy and farmers’ commitment constraints affect the waste recycling behavior of farmers; (ii) to focus on the impact of farmer’s commitment on sick and dead pigs waste management; (iii) to critically explore the impact mechanism of waste recycling behavior, its performance, and sustainable effectiveness from the perspective of the reward and punishment mechanism. The study will be crucial for formulating policies regarding dead and sick pig management.

## 2. Materials and Methods

### 2.1. Methods

The study’s prime aims are to evaluate the determinants of sick and dead pig waste recycling and to assess the moderating effects of rewards and punishment mechanisms undertaken by the Chinese government. However, the determinants are a set of factors or variables that can decisively affect the nature or outcome of a particular situation [48]. The determinants used in the study are taken from the existing literature. The study utilized factor analysis of the selected determinants. Factor analysis is a statistical approach for describing variance within associated variables from the perspective of a smaller number of unobserved variables known as factors [30]. It is mainly used to evaluate the structural validity of the questionnaire and find the relationships between the variables [49]. Therefore, the study used factor analysis to analyze the questionnaire’s seven basic variables, including the reward and punishment mechanism. Based on the existing literature [50,51], we have utilized six steps to perform the factor analysis, which are: (i) selecting and measuring a set of variables in a given domain, (ii) data screening in order to prepare the correlation matrix, (iii) factor extraction, (iv) factor rotation to increase interpretability, (v) interpretation, (vi) validation and reliability of the findings.

As the study assumes the explanatory variables (farmer’s decision) as dummy variables, and the question is answered with 0 and 1 (yes and no), it requires a particular regression model, namely the binary choice model (probit and logit) [30]. While statistically, probit and logit models have significant similarities, the two differ only in random error and the distribution of random error terms. Probit assumes that the random error term distribution obeys normal distribution, and the logit model assumes a logical distribution [52,53]. The probit model can solve the shortcomings of previous linear probability models in measuring binary selection in the linear probability model (LPM) [54]. At the same time, the logit model uses the cumulative distribution function of the logistic distribution [55]. On the other hand, probit models can be generalized to account for non-constant error variances in more advanced econometric settings (known as heteroskedastic probit models) and hence are widely used in some contexts by economists and political scientists [56]. Logit, also known as logistic regression, is more prevalent in health sciences such as epidemiology, partly because coefficients can be interpreted in odds ratios [57,58]. The study’s data confirm that the random error term is the normal distribution, and therefore we choose the probit model to craft our main findings. However, the probit coefficient cannot directly reflect the regression situation and compare the size of the coefficient, so marginal effect analysis has been employed to eliminate the shortcomings, as suggested by Chen et al. [54].

After choosing the appropriate model, the next step is to verify the findings with robustness testing. When certain parameters are changed, it is required to confirm whether the chosen methods and indicators can maintain a relatively consistent and stable interpretation [59,60]. The robustness test examines the validity of the evaluation methods and varifies the interpretation capabilities of the chosen index. Robustness testing has been highlighted as a benchmark for maintaining the consistency and accuracy of the outcomes [61,62,63]. Moreover, it is considered one of the vital quality assurance testing methodologies, highlighting the logical representation of any study’s findings [64]. This study used the logit model to verify the results as a robustness test framework.

**Figure 1:**
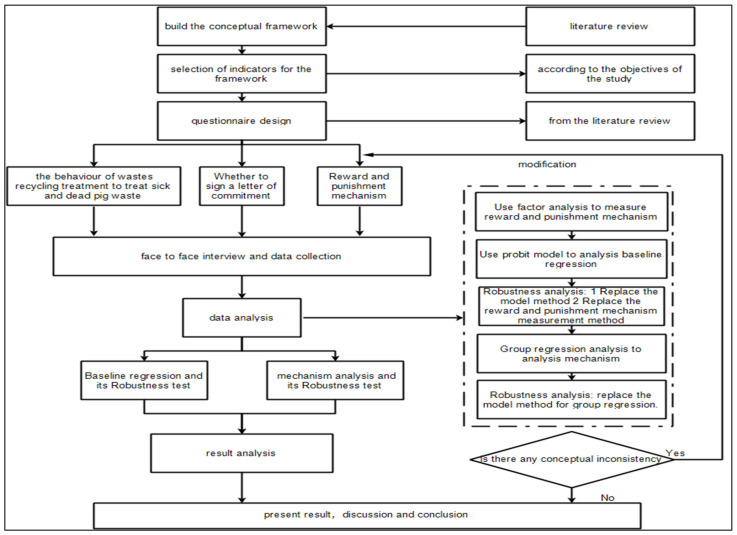
Adopted methodology of the study.

Interestingly, there is no uniform standard for performing robustness tests and no clear explanation about robustness testing procedures. Therefore, the standard of the robustness test varies significantly for each article according to its research purpose. In the existing literature, the commonly used methods of robustness testing are variable substitution, sample size change, sub-sample regression, supplementary variable, and model substitution methods [65,66,67]. We have adopted variable substitution and model substitution methods. The adopted methodology and its steps are shown in Figure 1.

### 2.2. Data Sources and Collections

The study uses a multistage sampling technique for selecting the farmers’ households. Multistage sampling divides large populations into several small stages to make the sampling process more practical, and it is considered a very effective tactic in the primary data collection process. It is mainly advantageous when data has to be collected from a geographically dispersed population and when face-to-face interviews are required [68]. First, the study purposively selected Henan, Shandong, and Hebei Provinces, representing three well-known major pig-raising provinces covering China’s central, northern, and eastern regions. The selected three provinces are the more famous pig-raising provinces in China, as shown in Table 1. Moreover, Henan, Shandong, and Hebei have implemented resource treatment mechanisms for managing sick and dead livestock and poultry. Moreover, the selected areas are situated in the regions where pigs are intensively raised, and the government has implemented reward and punishment mechanisms for safely managing sick and dead pig waste.

Shandong province is a coastal region of East China with a temperate climate, ranging between the humid subtropical and continental zones with four distinct seasons. Here, summers are hot and rainy (except for a few coastal areas), while the winter is cold and dry. Annual precipitation ranges from 550 to 950 mm, the vast majority of which occurs during summer due to monsoonal influences. It has around 1822 towns within 136 counties. Likewise, Hebei is a coastal province of North China with a monsoon-influenced humid continental climate, cold and dry winters, and hot and humid summers. Temperatures average −16 to −3 °C (3 to 27 °F) in January and 20 to 27 °C (68 to 81 °F) in July. The annual precipitation rates range from 400 to 800 mm (16 to 31), concentrated heavily in summer. It has around 2253 towns within 167 counties. Henan is a landlocked province of China, and it is situated in the central part of the country. Henan is situated in a humid subtropical zone and has a temperate climate. The province is situated south of the Yellow River and borders on a humid continental climate to the north. It has a distinct seasonal climate dominated by hot, humid summers due to the East Asian monsoon and generally cool, windy, and dry winters. Temperatures average around the freezing mark in January and 27 to 28 °C in July, with a great majority of the annual rainfall occurring during the summer. Henan Province consists of around 2453 towns in 158 counties.

However, Henan, Hebei, and Shandong have more prominent climatic advantages in pig raising. The climate is relatively mild, and there are relatively few accidents such as cold sickness and heatstroke in pig breeding. These three provinces have relatively sufficient water resources, fostering well-established pig breeding industries. Population distribution may have a significant influence on pig breeding. In densely populated areas, there are more laborers to support the breeding industries, and there is a tremendous market demand for pork. Recent statistics show that Shandong, Henan, and Hebei Province have 102 million, 99.4 million, and 74.6 million people, respectively. In addition, traditional eating habits and beliefs have profoundly affected the distribution pattern of China’s pig production industries. Henan, Hebei, and Shandong all have a rich agricultural culture, and their acceptance of pork is also relatively high.

**Figure 2:**
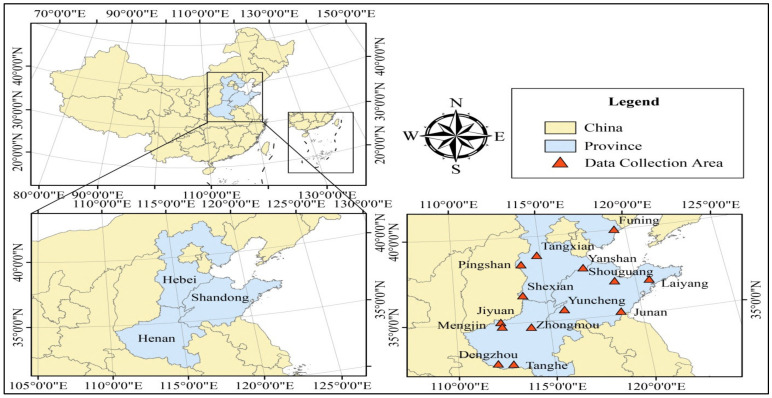
Study area Map.

In the second stage, around 3 to 5 counties or districts from each province were selected randomly and provided us with 14 counties in total. However, it accounts for five counties from 168 counties of Hebei province (Funing; 119°04′~119°46′ E, 39°41′~40°19′ N, Pingshan; 38°09′~38°45′ N, 113°31′~114°15′ E, Tangxian; 114°27′~115°03′ E, 38°37′~39°09′ N, Yanshan; 37°49′~38°06′ N, 116°56′~117°30′ E, Shexian; 36°17′~36°55′ N, 113°26′~114° E), five counties from 158 counties of Henan province (Jiyuan; 35°08′ N, 112°35′ E, Tanghe; 32°21′~32°55′ N, 112°28′~112°16′ E, Dengzhou; 32°22′~32°59′ N, 111°37′~111°20′ E, Mengjin; 112°12′~112°49′ E, 34°43′~34°57′ N, Zhongmou; 34°26′~34°56′ N, 113°46′~114°12′ E), and four counties from 168 counties of Shandong province (Yuncheng; 35°19′~35°52′ N, 115°40′~116°08′ E, Laiyang; 36°34′~37°9′ N, 120°31′~120°59′ E, Junan; 118°33′29″~119°06′06″ E, 35°00′15″~35°23′49″ N, Shouguang; 36°41′~37°19′ N, 118°32′~119°10′ E). Figure 2 represents the data collection area map.

In the third stage, we chose 14 counties or districts from Hebei (Funing, Pingshan, Tangxian, Yanshan, Shexian), Henan (Jiyuan, Tanghe, Dengzhou, Mengjin, Zhongmou), and Shandong (Yuncheng, Laiyang, Junan, Shouguang), respectively. After that, we selected 3 to 5 villages and towns with larger-scale pig breeding from the sample counties (districts) using systematic random sampling methods to ensure that the sample data gap in each province would not be too large. Finally, we have chosen 8–10 respondents from the selected villages and towns by employing systematic random sampling methods for the final data collection, which involves choosing the sample based on a regular interval rather than a fully random selection. It also helps the study ensure that the number of samples in each province is relatively similar. The survey was conducted in August–September 2018.

Before starting the formal survey, the investigators were trained on the relevant content of the questionnaire design. The respondents have been firmly briefed on all the relevant information and variables by the investigators to ensure the representativeness, accuracy, and reliability of the survey samples. Interestingly, because of the uneven distribution of pig farming regions and biosecurity considerations, there are certain restrictions on strangers entering the farm. Therefore, it was more challenging to select samples randomly. However, the research team members interviewed the town or township government leaders and the animal husbandry bureau to learn more about the development of the pig industry, the diseases of pigs, and the recycling of sick and dead pig waste in the surveyed area.

Furthermore, according to the situation, the project leader led the investigator directly to the pig farm to conduct face-to-face interviews with the respondents on the periphery of the farm. It helped the study to maximize the integrity, validity, and representativeness of the sample. It was inconvenient for the farmers to leave the farm for a small part of the questionnaires, so telephone interviews were used to conduct the questionnaire survey. The study only selected farmers with experience in handling dead and sick pigs, while 530 interviews were taken and 31 invalid samples were eliminated. Finally, 499 valid samples were obtained for further processing. Among them were 182 households in Hebei (87 households treated with waste recycling), 156 households in Henan (87 households treated with waste recycling), and 161 households in Shandong Province (56 households treated with waste recycling).

### 2.3. Variable Selection and Descriptive Statistical Analysis

#### 2.3.1. Explained Variable

The study used the popular statistical software, namely STATA (Stata Corp LLC, College Station, TX, USA) version 16.0, to craft the findings. The study chose farmers’ recycling behavior of sick and dead pig waste as the explained variable (dummy variable). A value of 1 is assigned if a farmer chose waste recycling treatment of their own sick and dead pigs, and the value is 0 if a farmer has not chosen waste recycling treatment. The survey samples found that 230 pig farmers tended to recycle sick and dead pig waste, and 269 pig farmers were not chosen for the recycle treatment.

#### 2.3.2. Core Explanatory Variables

The core explanatory variables of the study are: (i) farmers’ commitment, and (ii) reward and punishment mechanisms. Here, we chose whether to sign a commitment letter with the government for the waste recycling treatment of sick and dead livestock to reflect whether the farmers have committed. Among them, the signed commitment letter is assigned a value of 1, which means that the farmer has committed to recycling sick and dead pig waste. Similarly, the non-signed commitment is assigned a value of 0, which means that the breeder has not committed to recycling sick and dead pig waste. In the study, the reward and punishment mechanism has been calculated based on the extent of the governmental reward and punishment mechanism on the recycling of sick and dead pig wastes and their treatment behaviors. After the rotation mechanism, two common factors are obtained. Among them, the first common factor includes regulatory policies (does the regulatory policy for the harmless treatment of sick and dead pigs affect your handling of sick and dead pigs?) and punishment policies (does the punishment policy for improper handling behaviors, such as discarding sick and dead pigs, affect the handling of sick and dead pigs? Does the punishment policy on dealing with sick and dead pigs in the underground market affect your handling of sick and dead pigs?). This included (i) subsidy policy (does the subsidy policy for the recycling of sick and dead pig waste impact the handling of sick and dead pigs? Does the subsidy policy for the recycling treatment facility of sick and dead pigs impact the treatment of sick and dead pigs?), (ii) insurance policy (does the policy of linking waste recycling disposal and pig breeding insurance have an impact on your handling of sick and dead pigs?) and (iii) discount policy (does your behavior have an impact on the loan discount policy for the treatment of sick and dead pig waste in your home?). Finally, through factor analysis, comprehensive indicators of the punishment mechanism, reward mechanism, and reward and punishment mechanisms are obtained, respectively. As per the common factor analysis, the cumulative variance contribution rate is 74.938%, where the variance contribution rate of the first common factor is 39.563%, and the variance contribution rate of the second common factor is 35.375%. The specific indicators that reflect each dimension are shown in Table 2.

After the factor analysis of the reward and punishment mechanism, it was found that the Kaiser–Meyer–Olkin test (KMO) value was 0.689, and the Bartlett sphere test value was 2448.756 (*p*-value is 0.000), indicating that this sample data is suitable for factor analysis [69]. The formula for calculating the total index of the reward and punishment mechanism is:

*F* = {(39.563 × *F*_1_) + (35.375 × *F*_2_)}/74.938 (1)

Here, *F* represents the reward and punishment mechanism, *F*_1_ represents the reward mechanism, and *F*_2_ represents the punishment mechanism.

#### 2.3.3. Control Variable

Control or independent variables are the sorts of factors considered constant or limited terms in a research study [70]. They do not directly influence the study’s aims, but can control the main variables used in the study and influence the indirect outcomes [71]. They are also known as additional variables, which refer to potential factors or conditions that can affect the viability of the experiment [72]. These variables are used to alleviate the unidimensionality issues of the estimation [73,74] and may possess causal effects in multiple regression analysis [75].

The control variables selected in the article include household and family characteristics of farmers, environmental characteristics, and environmental protection awareness of farmers’ waste recycling treatment. Table 3 shows that the average age of the farmers in the sample is 47.904 years old, and most of them have junior high school or high school education. The pig breeding income accounts for 78.4% of the total family income. The average breeding scale is about 624 heads (number of pigs). To reflect environmental awareness of the recycling of sick and dead pig waste, the following questions have been asked: (i) is it possible to pollute the water body by improper handling, random burying and discarding of sick and dead pigs in the river? (ii) Is it possible to randomly bury the sick and dead pigs and cause soil pollution with heavy metals and residual antibiotics due to improper handling? (iii) Is it possible to cause air pollution by improper handling or open burning of sick and dead pigs? The average values of these three variables are 3.385, 3.427, and 3.361, indicating that the survey sample farmers have a high level of environmental awareness about the recycling of sick and dead pig waste, as suggested by Oliver et al. [76]. Most farmers understand that improper handling of sick and dead pigs will cause damage to the environment.

### 2.4. Empirical Model Setting

In order to investigate the impact of commitment, reward, and punishment mechanisms on the recycling behavior of sick and dead pig waste, this paper sets up the following model, where Y represents the recycling treatment behavior of the farmer’s sick and dead pig waste in the formula. Among them, the value of 1 is assigned to the waste recycling treatment of their own sick and dead pigs, and the value of 0 is not selected for the waste recycling treatment of their own sick and dead pigs. Since the dependent variable is a dummy variable, this paper adopts the probit model for empirical estimation. The general form of the model can be expressed as follows:

(2) $$Y^{*}=\beta_{0}+\beta_{1}\mathrm{Commitment}+\beta_{2}F+\beta_{3}X+\beta_{4}\mathrm{Province}+\xi$$

(3) $$Y^{*}=\beta_{0}+\beta_{1}\mathrm{Commitment}+\beta_{2}F_{1}+\beta_{3}F_{2}+\beta_{4}X+\beta_{5}\mathrm{Province}+\xi$$

**Figure 3:**
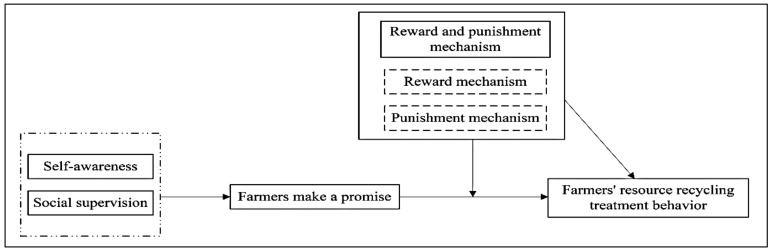
Theoretical framework of the study.

Among them Y^*^ is the latent variable, *β_0_*, *β_1_*,…,*β_4_* as the coefficient to be estimated, the residual term obeys the normal distribution, and the variance is *σ^2^*, it is *ζ~N*(0,*σ^2^*). Among the explanatory variables, commitment indicates whether the farmer has committed; *F*, *F*_1_, *F*_2_ are the reward and punishment mechanism, reward mechanism, and punishment mechanism, respectively. These four variables are the core explanatory variables. Seemingly, *X* represents a vector of control variables, including household head characteristics, environmental characteristics, and environmental protection awareness of farmers’ waste recycling treatment. The specific mechanism analysis framework is shown in Figure 3.

## 3. Results

### 3.1. The Impact of Whether to Sign a Letter of Commitment

#### 3.1.1. Baseline Regression

Shown in Table 4, column (1) are the two dimensions of the reward and punishment mechanism and the impact of commitments on the recycling of sick and dead pig waste. Column (2) is the reward and punishment mechanism and the impact of farmers’ commitments to recycling sick and dead pig waste. Column (3) reports the marginal effect analysis of the column (1) model. Column (4) separately reports the marginal effect analysis of column (2).

Since the probit model cannot provide intuitive quantitative meanings, it only contains information about the statistical significance of the explanatory variables and the direction of action, the degree of influence of each explanatory variable on the dependent variable is not obtained, and the marginal effect of each variable needs to be calculated. As shown in Table 4, for column (1) and column (3), the estimated coefficients of incentive mechanism, punishment mechanism, and commitment are significant at the levels of 1%, 1%, and 5%, and they have significant positive effects on the resource treatment behavior of farmers’ sick and dead pig waste as recommended by Min et al. [77]. The marginal effect test results showed that with every 0.1 increase in the possibility of farmers committing, the probability of recycling treatment of dead pig waste increased by 0.998%. When the reward mechanism index increased by 0.1, the probability of recycling sick and dead pig waste increased by 1.13%. When the punishment mechanism index increased by 0.1, the probability of recycling sick and dead pig waste increased by 0.898%. The estimated coefficients of the reward and punishment mechanism and commitment were significant at the level of 1% and 5%, and they had a significant positive impact on the recycling treatment behavior of livestock waste. The results of the marginal effect test showed that for every 0.1 increase in the probability of a farmer making a farmer’s commitment, the probability of the occurrence of diseased and dead pig waste recycling treatment behavior increased by 0.959%. Every time the reward and punishment mechanism index increases by 0.1, the probability of resource treatment of sick and dead pig waste increases by 2.046%. It can be seen that the reward and punishment mechanism for driving farmers’ commitments and government policies can effectively promote the resource treatment of sick and dead pig waste by farmers.

Among the control variables, Is anyone in the family a village cadre, the proportion of the farming labor force in the total population, and the pig-raising scale variables are significantly positive. This shows that there are people in the family who are village cadres, and the more farming laborers account for the total population and the larger the number of farms, the more the farmer tends to make decisions about the recycling of sick and dead pig waste. Whether the variable of setting a collection point for sick and dead pigs is significantly positive indicates that there is a collection point for sick and dead pigs in the area where the farmers are located, and the farmers in this area are more inclined to make decisions about the recycling of sick and dead pig waste. The variable “Is improper handling likely to cause water pollution by burying and discarding sick and dead pigs in the river at will?” reflects the farmers’ awareness of the environmental impact caused by improper handling of sick and dead pigs. This variable is significantly positive, indicating that the higher the farmers’ awareness, the more likely they will recycle sick and dead pig waste. In addition, the regional dummy variables are more significant, indicating significant regional differences in the implementation of the recycling of sick and dead pig waste.

#### 3.1.2. Robustness Test

In order to test the robustness of the above-mentioned empirical analysis results, this paper conducts the robustness test of the regression results in Table 4 by replacing the model form and changing the measurement method of core variables.

The probit model involved in the equation was replaced with the logit model, and the robustness test was performed by changing the distribution form of the data. The results are shown in Table 5. It can be found that after changing the model setting method, the results are consistent, regardless of the significance of the variable or the sign of the coefficient. The robustness test results all support the positive and significant impact of the government’s signing of a letter of commitment, the reward and punishment mechanism and its two dimensions on the recycling of sick and dead pig waste. The previous research conclusions are still valid.

In the study, the variables measured by the reward and punishment mechanism are added together to obtain the total score, acting as the policy effect. We use this indicator to re-regress, as shown in the results of columns 3 and 4 in Table 5. The regression results in Table 4 are consistent with the reward and punishment mechanism and its two-dimensional measurement methods. The robustness test of the study also found that the government’s signing of a letter of commitment and the reward, and punishment mechanism positively and significantly impact the recycling of sick and dead pig waste. Therefore the research conclusions are valid, and the research conclusions of this article are robust.

### 3.2. Analysis of the Credible Farmer’s Commitment Mechanism

In order to examine whether the reward and punishment mechanism has committed to influencing the recycling treatment behavior of sick and dead pig waste, the study uses the group regression method. Moreover, it is also used to test whether the farmers commit to the recycling disposal of sick and dead pig waste under the condition that the reward and punishment mechanism and its two dimensions have different levels of influence. Columns (1) and (2) in Table 6 respectively reflect the impact of whether or not commitments are made on the recycling treatment of sick and dead pig waste under the influence of different incentive mechanisms. The results show that, under the influence of a low-level reward mechanism (*F*_1_ < 0), whether the farmers commit to implementing the decision on the recycling of sick and dead pig waste has no significant impact, and the significance test is not passed. However, under the influence of a high-level reward mechanism (*F*_1_ ≥ 0), whether the farmers commit to implementing their decisions on the recycling of sick and dead pig waste has a significant positive impact, and the significance level is 1%.

In the same way, columns (3) and (4) respectively reflect the impact of whether or not commitments are made to the recycling of sick and dead pig waste under the influence of different punishment mechanisms. The results show that, under the influence of the low-level punishment mechanism (*F*_2_ < 0), whether the farmers commit to their implementation of the decision to treat sick and dead pig waste as recycling does not have a significant impact and fails the significance test. Under the influence of a high-level punishment mechanism (*F*_2_ ≥ 0), whether the farmers commit to implementing the decision on the recycling of sick and dead pig waste has a significant positive impact, and the significance level is 1%. Columns (5) and (6) respectively reflect the impact of whether commitments are made on the recycling treatment of sick and dead pig waste under the influence of different reward and punishment mechanisms. The results show that under the influence of the low-level reward and punishment mechanism (F < 0), whether the farmers make a commitment does not have a significant impact on the implementation of their decisions on the recycling of sick and dead pig waste, and they have not passed the significance test. Under the influence of a high-level reward and punishment mechanism (F ≥ 0), whether the farmers commit to implementing their decision on the recycling of sick and dead pig waste has a significant positive impact, and the significance level is 1%.

The above analysis shows that the reward and punishment mechanism and its two dimensions have enhanced the impact of farmers’commitments to the recycling of sick and dead pig waste. This could be because farmers can have a good reward and punishment environment when the reward and punishment mechanism is highly influenced. Therefore, it is conducive to playing informal institutional norms such as commitments and autonomous driving in this environment. At the same time, with the incentive and supervision of external formal systems such as reward and punishment policies, it is helpful for farmers to make decisions about the recycling of sick and dead pig waste. This will further improve the efficiency of implementing commitments and achieve a win–win situation for the effective recovery of sick and dead livestock and poultry carcasses, and rural ecological and environmental protection.

Table 7 represents the robustness test of the above-mentioned empirical results. The study uses regression analysis by replacing the forms of the model. The comparison of the results presented in Table 6 and Table 7 reflects a consistent outcome regarding the significance and coefficient signs of the main variables. Moreover, the robustness test also confirms that the reward and punishment mechanism has specific moderating effects on whether to commit or not to recycle sick and dead pig waste.

## 4. Discussion

The recycling and safe treatment of sick and dead pig waste is a typical public health and safety behavioral activity that is shaped by various externalities such as cost, the criticality of the process, farmers’ moral views and public perceptions, and therefore its implementation process requires the active participation of farmers [78]. From the perspective of public economics, this behavior is recognized as pro-environmental behavior, which can influence the behavior of farmers to a greater extent as it fosters public attributes, although it contradicts farmers’ personal interests [79]. It usually takes extra effort and resources for farmers to treat and recycle dead pig carcasses. However, it is beneficial to the environment and reduces pollution in the long run. In addition, government supervision is more complex and often faces free-riding issues. Therefore, the relevant departments need to adopt stricter reward and punishment policies to prevent farmers from improperly handling sick and dead pigs. They should try to reduce external constraints and introduce easy and simple governmental interventions to encourage farmers towards adequate recycling treatment behaviors [80]. The study found a strong correlation between environmental regulation, economic incentive policies, and reward and punishment mechanisms among the surveyed pig farmers. The outcome is in parallel with the findings of LiMei and YaQing [81]. In a study of Suburban areas of Hanoi Capital, Vietnam, Duong et al. [82] have found that the reward and punishment mechanism significantly impacts environmentally friendly behaviors such as the eco-friendly recycling of sick and dead pig waste. The study also comprises similar results.

In addition, some scholars have found that farmers’ commitment has a profound link to promoting environmentally friendly behaviors among themselves. In this process, the Chinese government also formulated more practical reward and punishment policies to encourage farmers to adopt waste recycling and resource treatment behaviors [10]. Farmers voluntarily sign a commitment letter with the village committee for the recycling of sick and dead pig waste, in which they will inform the farmers of the consequences of improper handling of livestock and poultry carcasses, and they will voluntarily post the commitment letter in the most conspicuous position of the farm [83]. Therefore, the governmental authority should sign a waste recycling treatment commitment letter with the farmers to clarify the rights and obligations of both parties. On the one hand, out of environmental protection awareness of, the farmers commit to correct and harmless treatment methods when discovering that their livestock are infected with diseases, and adopt self-restraint, standardization, and supervision [84]. The study found that the reward and punishment mechanism is more effective than the farmers’ commitment through the marginal effect analysis. In addition, mechanism studies have found that commitments can only play a role when the reward and punishment mechanism works well, ultimately making the commitment credible.

However, the farmer’s commitment is a voluntary behavior derived from the farmer’s moral views, social norms, and social supervision. The social network formed by the village’s neighborhood urges farmers to make commitments and helps to build social norms and social supervision [85]. Interestingly, due to the lack of legal support, the extent of farmers’ commitments may not be adequate in several regions of the world, especially in developing economies [86]. The study found that farmers ignore their previous commitments without external pressure and economic incentives. However, existing studies showed that the effective implementation of the reward and punishment mechanism could guarantee the continuity of commitments and supported the findings [87,88]. According to Lu et al. [89], if the reward and punishment mechanism works well, it can lead to practical and adequate commitment from farmers. This study also found similar findings and highlighted a crucial link among the credible commitments, rewards, and punishments, and together they have promoted environmentally friendly behavior among farmers. Moreover, the study also found that farmers’ initiative in resource utilization can lead to cost savings and improve the profit margin, which eventually helps them to foster more environmental consciousness; the findings are supported by Wąs et al. [90].

## 5. Conclusions

The study uses factor analysis, probit model, and adjustment effect analysis to critically evaluate the relationship between commitment constraints, reward and punishment mechanisms, and recycling behavior of sick and dead pig wastes. We studied the findings based on the micro-survey data of 530 pig farmers in 14 counties of Henan, Hebei, and Shandong provinces, China. The study finds that both the reward and punishment mechanism and farmers’ commitments significantly impact farmers’ recycling treatment of sick and dead pig waste. Among them, the reward and punishment mechanism has a more substantial effect than the farmer’s commitment. According to the marginal effects, every time a farmer’s commitment to utilizing safer waste treatment increases by 0.1, the probability of disease and waste resource treatment efficiency will increase by 0.959%. When the reward and punishment mechanism index increases by 0.1, eventually the probability of resource treatment of sick and dead pig waste increases by 2.046%. Therefore, it can be estimated that the incentive mechanism is more effective than the penalty mechanism. Every time the incentive mechanism index increases by 0.1, the probability of sick and dead pig waste recycling treatment behavior will increase by 1.13%. Similarly, when the penalty mechanism index increases by 0.1, the probability of diseased and dead pig waste resource treatment will increase by 0.898%. According to the adjustment effect, the reward and punishment mechanism greatly influences the farmers’ commitment and eventually fosters a significant impact on farmers’ decision-making processes for recycling sick and dead pig waste. Seemingly, when it has a low degree of influence, commitment possesses an insignificant influence on the decision-making process. The results indicate that the reward and punishment mechanisms directly link to enhancing the impact of the farmers’ commitments on recycling behavior. In summary, it can assume that, if the reward and punishment mechanism works well, it can strengthen the farmers’ commitments.

There are also some shortcomings in the study. First, the continuity of commitments is a dynamic process and it requires continuous-time testing. Although the article proves that reward and punishment mechanisms and commitment are conducive to encouraging the farmers to make decisions on the recycling of sick and dead pig waste, the reward and punishment mechanism is conducive to the effect of commitments. However, the data and research areas are limited and fail to reflect the farmers’ commitment to continuous impact. Secondly, the article explores the influence mechanism of commitment through self-cognition and social supervision in theory. However, the data obtained in the study are limited, and no in-depth empirical research is carried out on it. Therefore, the results of the study may have specific limitations and are not refect generalized views for the whole country. In the future, scholars can further study the impact of farmers’ commitments on the recycling of sick and dead pig waste in the context of different policies and socio-economic characteristics. Interestingly, in-depth discussions should also be conducted regarding how to encourage farmers to decide on the recycling of sick and dead pig waste and how commitments can continue to take effect.

## Figures and Tables

**Table 1:** The pig breeding ranking of the selected provinces.

Years	Henan		Hebei		Shandong	
	The Number of Pigs Slaughtered	Rank	The Number of Pigs Slaughtered	Rank	The Number of Pigs Slaughtered	Rank
2013	5997	2	3452	8	4798	4
2014	6310	2	3638	7	4955	4
2015	6171	2	3551	7	4836	4
2016	6005	2	3434	7	4662	4
2017	6220	2	3785	7	5181	4

Unit: Ten thousand. Source: China Statistical Yearbook, available online: http://www.stats.gov.cn/english/Statisticaldata/AnnualData/ (accessed on 2 July 2021).

**Table 2:** Descriptive statistics of the study.

Variable		Description	Mean	Std.
Punishment mechanism	Regulatory policy	Does the regulatory policy for the harmless disposal of sick and dead pigs affect your family’s handling of sick and dead pigs? 1 = No effect at all, 2 = No effect, 3 = Normal, 4 = Influence, 5 = Great influence	3.521	1.063
	Penalty policy	Does the punishment policy for improper handlings, such as the discarding of sick and dead pigs, affect the handling of sick and dead pigs? 1 = No effect at all, 2 = No effect, 3 = Normal, 4 = Influence, 5 = Great influence	3.565	0.962
	Punishment policy	Does the policy of cracking down on the trading behavior of sick and dead pigs in the underground market have any impact on your family’s handling of sick and dead pigs? 1 = No effect at all, 2 = No effect, 3 = Normal, 4 = Influence, 5 = Great influence	3.711	0.737
Reward Mechanism	Subsidy policy	Does the subsidy policy for the recycling of sick and dead pig waste impact your family’s handling of sick and dead pigs? 1 = No effect at all, 2 = No effect, 3 = Normal, 4 = Influence, 5 = Great influence	3.196	1.215
	Insurance policy	Does the policy linking waste recycling-based treatment and pig breeding insurance impact the handling of sick and dead pigs in your family? 1 = No effect at all, 2 = No effect, 3 = Normal, 4 = Influence, 5 = Great influence	3.190	1.246
	Subsidy policy	Does the subsidy policy for the waste recycling treatment facility of sick and dead pigs have any impact on your family’s handling of sick and dead pigs? 1 = No effect at all, 2 = No effect, 3 = Normal, 4 = Influence, 5 = Great influence	2.483	1.342
	Discount policy	Does the loan interest discount policy for the waste recycling treatment facility of sick and dead pigs have any impact on your family’s handling of sick and dead pigs? 1 = No effect at all, 2 = No effect, 3 = Normal, 4 = Influence, 5 = Great influence	2.381	1.323

**Table 3:** Descriptive statistical analysis.

Variable	Description	Mean	Std.
Whether to adopt waste recycling treatment to treat sick and dead pig waste	1 = yes; 0 = no	0.461	0.499
Whether to sign a letter of commitment	1 = yes; 0 = no	0.707	0.455
*F*_1_	Factor analysis	0.000	1.000
*F*_2_	Factor analysis	0.000	1.000
*F*	Factor analysis	0.000	0.708
Age	Age of head of household, unit: years	47.904	8.614
Education level	Education level of the head of household, unit: year	9.066	2.832
Farming years	Unit: Year	9.166	5.652
Village cadre	Is anyone in the family a village cadre? 1 = Yes 0 = No	0.196	0.398
The proportion of farming labor force in total population	Number of pig-raising labor force/total population in household	0.508	0.238
The proportion of pig income in total income	Income from pig raising/all income earned in a year	0.784	0.213
Farming scale	Number of pigs	624.16	1507.95
Whether to set up a collection point for sick and dead pigs	1 = Yes 0 = No	0.447	0.498
Is improper handling likely to cause water pollution by burying and discarding sick and dead pigs in the river at will?	1 = completely impossible, 2 = impossible, 3 = general, 4 = possible, 5 = very likely	3.385	0.665
Is improper handling of sick and dead pigs likely to cause soil pollution with heavy metals and residual antibiotics?	1 = completely impossible, 2 = impossible, 3 = general, 4 = possible, 5 = very likely	3.427	1.135
Is it likely that improper handling of sick and dead pigs will cause air pollution?	1 = completely impossible, 2 = impossible, 3 = general, 4 = possible, 5 = very likely	3.361	1.176
The convenience of obtaining waste recycling equipment.	1 = very inconvenient, 2 = inconvenient, 3 = general, 4 = convenient, 5 = very convenient	2.659	1.202

**Table 4:** The impact of whether to sign a letter of commitment and the reward and punishment mechanism on the behavior of farmers in adopting the waste recycling treatment of sick and dead pigs.

	Probit		Marginal Effect	
	(1)	(2)	(3)	(4)
Whether to sign a letter of commitment	0.4806 ** (0.2120)	0.4615 ** (0.2077)	0.0998 ** (0.0435)	0.0959 ** (0.0427)
*F*_1_	0.5440 *** (0.1113)		0.1130 *** (0.0211)	
*F*_2_	0.4320 *** (0.1121)		0.0898 *** (0.0221)	
*F*		0.9843 *** (0.1858)		0.2046 *** (0.0347)
age	−0.0108 (0.0125)	−0.0103 (0.0124)	−0.0022 (0.0026)	−0.0021 (0.0026)
education level	−0.0137 (0.0357)	−0.0120 (0.0355)	−0.0029 (0.0074)	−0.0025 (0.0074)
Farming years	0.0222 (0.0202)	0.0229 (0.0201)	0.0046 (0.0042)	0.0048 (0.0042)
Village cadre	0.6639 *** (0.2064)	0.6601 *** (0.2065)	0.1379 *** (0.0412)	0.1372 *** (0.0413)
Proportion of farming labor force in total population	1.2168 *** (0.4308)	1.1909 *** (0.4271)	0.2528 *** (0.0878)	0.2476 *** (0.0871)
Proportion of pig income in total income	−0.6980 (0.4997)	−0.6700 (0.4968)	−0.1450 (0.1037)	−0.1393 (0.1031)
Farming scale	0.0013 *** (0.0002)	0.0013 *** (0.0002)	0.0003 *** (0.0000)	0.0003 *** (0.0000)
Whether to set up a collection point for sick and dead pigs	0.9533 *** (0.2058)	0.9347 *** (0.2017)	0.1981 *** (0.0393)	0.1943 *** (0.0385)
Is improper handling likely to cause water pollution by burying and discarding sick and dead pigs in the river at will?	0.2305 * (0.1342)	0.2302 * (0.1344)	0.0479 * (0.0276)	0.0479 * (0.0276)
Is improper handling of sick and dead pigs likely to cause soil pollution with heavy metals and residual antibiotics?	−0.0571 (0.1257)	−0.0492 (0.1245)	−0.0119 (0.0261)	−0.0102 (0.0259)
Is it likely that improper handling of sick and dead pigs will cause air pollution?	0.1509 (0.1211)	0.1527 (0.1211)	0.0313 (0.0250)	0.0317 (0.0250)
The convenience of obtaining waste recycling equipment.	0.0510 (0.0924)	0.0528 (0.0922)	0.0106 (0.0192)	0.0110 (0.0192)
Is it Henan	0.4734 ** (0.2120)	0.4728 ** (0.2119)	0.0984 ** (0.0432)	0.0983 ** (0.0433)
Is it Hebei	0.2519 (0.2300)	0.2343 (0.2266)	0.0523 (0.0477)	0.0487 (0.0470)
*N*	499	499	499	499
LR chi2	320.45	320.23	320.45	320.23
Prob > chi2	0.0000	0.0000	0.0000	0.0000
Pseudo R^2^	0.4653	0.4650	0.4653	0.4650

Note: Standard errors in parentheses and * *p* < 0.10, ** *p* < 0.05, *** *p* < 0.01.

**Table 5:** Robustness analysis.

	Change Model Settings		Change the Measurement Method of Reward and Punishment Mechanism	
	(1)	(2)	(3)	(4)
Whether to sign a letter of commitment	0.9385 ** (0.3763)	0.9020 ** (0.3690)	0.5268 ** (0.2099)	0.5035 ** (0.2063)
*F*_1_	0.9246 *** (0.2021)		0.1247 *** (0.0259)	
*F*_2_	0.7216 *** (0.2017)		0.0948 ** (0.0428)	
ALL		1.6627 *** (0.3414)	-	0.1170 *** (0.0225)
Control variable	Control	Control	Control	Control
Province variable	Control	Control	Control	Control
_cons	−5.3375 *** (2.0058)	−5.4465 *** (1.9945)	−5.5054 *** (1.2648)	−5.7457 *** (1.2058)
*N*	499	499	499	499
LR chi2	321.42	321.16	318.91	318.54
Prob > chi2	0.000	0.000	0.000	0.000
Pseudo R^2^	0.4667	0.4663	0.4631	0.4625

Note: Standard errors in parentheses and ** *p* < 0.05, *** *p* < 0.01.

**Table 6:** The impact of whether commitments are made under the influence of different reward and punishment mechanisms on the recycling of sick and dead pig waste.

	*F*_1_ ≥ 0	*F*_1_ < 0	*F*_2_ ≥ 0	*F*_2_ < 0	ALL ≥ 0	ALL < 0
	(1)	(2)	(3)	(4)	(5)	(6)
Whether to sign a letter of commitment	1.2085 *** (0.3000)	0.3956 (0.3554)	1.1990 *** (0.3092)	0.1998 (0.3743)	1.0564 *** (0.2731)	−0.8419 (0.5297)
Control variable	Control	Control	Control	Control	Control	Control
Province variable	Control	Control	Control	Control	Control	Control
_cons	−0.6257 (1.8052)	−4.5257 ** (1.8188)	−4.6711 *** (1.6174)	−2.0995 (1.9602)	−1.9489 (1.5355)	−7.3251 ** (3.2509)
*N*	223	276	277	222	294	205
LR chi2	104.68	182.91	124.73	145.67	123.58	87.51
Prob > chi2	0.0000	0.0000	0.0000	0.0000	0.0000	0.0000
Pseudo R^2^	0.3642	0.5392	0.3393	0.5712	0.3412	0.5612

Note: Standard errors in parentheses and ** *p* < 0.05, *** *p* < 0.01.

**Table 7:** Robustness test of the mechanism analysis.

	*F*_1_ ≥ 0	*F*_1_ < 0	*F*_2_ ≥ 0	*F*_2_ < 0	ALL ≥ 0	ALL < 0
Whether to sign a letter of commitment	2.2432 *** (0.5692)	0.9116 (0.6447)	2.2873 *** (0.5592)	0.2798 (0.7759)	2.0463 *** (0.5127)	−1.5606 (1.0081)
Control variable	Control	Control	Control	Control	Control	Control
Province variable	Control	Control	Control	Control	Control	Control
_cons	0.1286 (3.2631)	−8.4311 *** (3.2489)	−8.4336 *** (2.8480)	−3.6985 (3.6932)	−2.7981 (2.8412)	−12.6172 ** (5.7450)
*N*	223	276	277	222	294	205
LR chi2	105.73	183.32	130.19	145.63	129.23	86.66
Prob > chi2	0.0000	0.0000	0.0000	0.0000	0.0000	0.0000
Pseudo R^2^	0.3678	0.5404	0.3541	0.5711	0.3568	0.5557

Note: Standard errors in parentheses and ** *p* < 0.05, *** *p* < 0.01.

## Data Availability

The associated dataset of the study is available upon request to the corresponding author.

## References

[1] Sud D., Mahajan G., Kaur M.P. (2008). Agricultural Waste Material as Potential Adsorbent for Sequestering Heavy Metal Ions from Aqueous Solutions—A Review. Bioresour. Technol..

[2] Mason-D’Croz D., Bogard J.R., Herrero M., Robinson S., Sulser T.B., Wiebe K., Willenbockel D., Godfray H.C.J. (2020). Modelling the Global Economic Consequences of a Major African Swine Fever Outbreak in China. Nat. Food.

[3] Wu L., Xu G., Li Q., Hou B., Hu W., Wang J. (2017). Investigation of the Disposal of Dead Pigs by Pig Farmers in Mainland China by Simulation Experiment. Environ. Sci. Pollut. Res..

[4] Qian Y., Song K., Hu T., Ying T. (2018). Environmental Status of Livestock and Poultry Sectors in China under Current Transformation Stage. Sci. Total Environ..

[5] Chen X., Qiu G., Wu L., Xu G., Wang J., Hu W. (2017). Influential Impacts of Combined Government Policies for Safe Disposal of Dead Pigs on Farmer Behavior. Environ. Sci. Pollut. Res..

[6] Guidoni L.L.C., Martins G.A., Guevara M.F., Brandalise J.N., Lucia T., Gerber M.D., Corrêa L.B., Corrêa É.K. (2021). Full-Scale Composting of Different Mixtures with Meal from Dead Pigs: Process Monitoring, Compost Quality and Toxicity. Waste Biomass Valor..

[7] Zheng J.-L., Zhu M.-Q., Wu H. (2015). Alkaline Hydrothermal Liquefaction of Swine Carcasses to Bio-Oil. Waste Manag..

[8] Gwyther C.L., Williams A.P., Golyshin P.N., Edwards-Jones G., Jones D.L. (2011). The Environmental and Biosecurity Characteristics of Livestock Carcass Disposal Methods: A Review. Waste Manag..

[9] Sun C., Wu H. (2013). Assessment of Pollution from Livestock and Poultry Breeding in China. Int. J. Environ. Stud..

[10] Si R., Wang M., Lu Q., Zhang S. (2020). Assessing Impact of Risk Perception and Environmental Regulation on Household Carcass Waste Recycling Behaviour in China. Waste Manag. Res..

[11] Sander J.E., Warbington M.C., Myers L.M. (2002). Selected Methods of Animal Carcass Disposal. J. Am. Vet. Med. Assoc..

[12] Li F., Cheng S., Yu H., Yang D. (2016). Waste from Livestock and Poultry Breeding and Its Potential Assessment of Biogas Energy in Rural China. J. Clean. Prod..

[13] Glanville T.D., Ahn H.K., Richard T.L., Shiers L.E., Harmon J.D. (2009). Soil Contamination Caused by Emergency Bio-Reduction of Catastrophic Livestock Mortalities. Water Air Soil. Pollut..

[14] Gwyther C.L., Jones D.L., Golyshin P.N., Edwards-Jones G., Williams A.P. (2012). Fate of Pathogens in a Simulated Bioreduction System for Livestock Carcasses. Waste Manag..

[15] Jian W., Chen Y., Jian T. (2019). Farmers’ loss aversion and treatment of dead hogs: An investigation of 404 hog farmers. China Rural. Econ..

[16] Pandey P., Vidyarthi S.K., Vaddella V., Venkitasamy C., Pitesky M., Weimer B., Pires A.F.A. (2020). Improving Biosecurity Procedures to Minimize the Risk of Spreading Pathogenic Infections Agents After Carcass Recycling. Front. Microbiol..

[17] Li P.J. (2009). Exponential Growth, Animal Welfare, Environmental and Food Safety Impact: The Case of China’s Livestock Production. J. Agric. Environ. Ethics.

[18] Taiganides E.P. (1992). Pig Waste Management and Recycling: The Singapore Experience.

[19] Kaufmann T. (2015). Sustainable Livestock Production: Low Emission Farm—The Innovative Combination of Nutrient, Emission and Waste Management with Special Emphasis on Chinese Pig Production. Anim. Nutr..

[20] Sarkar A., Wang H., Rahman A., Qian L., Memon W.H. (2022). Evaluating the Roles of the Farmer’s Cooperative for Fostering Environmentally Friendly Production Technologies-a Case of Kiwi-Fruit Farmers in Meixian, China. J. Environ. Manag..

[21] Wu L., Xu G., Wang X. (2016). Identifying Critical Factors Influencing the Disposal of Dead Pigs by Farmers in China. Environ. Sci Pollut Res..

[22] Hennessy D.A., Wolf C.A. (2018). Asymmetric Information, Externalities and Incentives in Animal Disease Prevention and Control. J. Agric. Econ..

[23] Vu T.K.V., Tran M.T., Dang T.T.S. (2007). A Survey of Manure Management on Pig Farms in Northern Vietnam. Livest. Sci..

[24] Martín-Hernández E., Martín M., Ruiz-Mercado G.J. (2021). A Geospatial Environmental and Techno-Economic Framework for Sustainable Phosphorus Management at Livestock Facilities. Resour. Conserv. Recycl..

[25] XiangHai M., JunBiao Z., Peng L., XiaoKun C. (2014). Summary of livestock environmental pollution and environmental management policies. J. Ecol. Rural Environ..

[26] Terry J.P., Khatri K. (2009). People, Pigs and Pollution—Experiences with Applying Participatory Learning and Action (PLA) Methodology to Identify Problems of Pig-Waste Management at the Village Level in Fiji. J. Clean. Prod..

[27] Ferreira L., Duarte E., Figueiredo D. (2012). Utilization of Wasted Sardine Oil as Co-Substrate with Pig Slurry for Biogas Production—A Pilot Experience of Decentralized Industrial Organic Waste Management in a Portuguese Pig Farm. Bioresour. Technol..

[28] Ostrom E., Schroeder L., Wynne S. (1993). Institutional Incentives and Sustainable Development: Infrastructure Policies in Perspective.

[29] Kadurumba C., Nwankwo E.S., Ene O.J., Kadurumba O.E. (2020). Analysis of Waste Management and Profit Efficiency in Pig Production in Owerri Agricultural Zone of Imo State. Niger. J. Anim. Sci. Technol..

[30] Weible D., Christoph-Schulz I., Salamon P., Zander K. (2016). Citizens’ Perception of Modern Pig Production in Germany: A Mixed-Method Research Approach. Br. Food J..

[31] Webb J., Broomfield M., Jones S., Donovan B. (2014). Ammonia and Odour Emissions from UK Pig Farms and Nitrogen Leaching from Outdoor Pig Production. A Review. Sci. Total Environ..

[32] Hutchings N.J., ten Hoeve M., Jensen R., Bruun S., Søtoft L.F. (2013). Modelling the Potential of Slurry Management Technologies to Reduce the Constraints of Environmental Legislation on Pig Production. J. Environ. Manag..

[33] Jinzhi Z., Long L., Da Y., Jiaxue L., Shaojun Z., Yuping Y., Jie Z., Yushuang L. (2017). Application of Comprehensive Harmless Waste Treatment Technology to Treat Drilling Cuttings in the Tian Mountain Front Block in Tarim Basin. OnePetro.

[34] Ego A., Samuel N. (2013). Jervas Statutory Regulations of Dead Animal Carcass Disposal in Nigeria: A Case Study of Enugu State. AJAR.

[35] Ji B., Du J., Qi H., Peng Y., Zhu R., Wu C., Wang Z. (2021). Harmless Treatment and Comprehensive Utilization of Dairy Farming Waste Based on Artificial Intelligence. J. Phys. Conf. Ser..

[36] Cao H., Zhu X., Heijman W., Zhao K. (2020). The Impact of Land Transfer and Farmers’ Knowledge of Farmland Protection Policy on pro-Environmental Agricultural Practices: The Case of Straw Return to Fields in Ningxia, China. J. Clean. Prod..

[37] Rezaei-Moghaddam K., Vatankhah N., Ajili A. (2020). Adoption of Pro-Environmental Behaviors among Farmers: Application of Value–Belief–Norm Theory. Chem. Biol. Technol. Agric..

[38] Wang Z., Jiang Y., Wang S., Zhang Y., Hu Y., Hu Z., Wu G., Zhan X. (2020). Impact of Total Solids Content on Anaerobic Co-Digestion of Pig Manure and Food Waste: Insights into Shifting of the Methanogenic Pathway. Waste Manag..

[39] Hua L., Xun H., Xi G., Fang L., Chang D. Pollution and harmless treatment of livestock manure and technology and policy of organic fertilizer in China. Proceedings of the China’s Modern Agricultural Development Forum in 2014.

[40] YuJun S., LiXin Z., HaiBo M. (2013). Present status of harmless disposal of dead livestock and poultry in China and counter-measures. J. Agric. Sci. Technol..

[41] Wang B., Huang Y., Liu W., Chen S., Zhu J., Belzile N., Chen Y.-W., Liu M., Liu C. (2021). Returning Excrement from Livestock, Poultry, and Humans to Farmland as Nutrient Resources for Crop Growth: Assessment of Rural China. Process. Saf. Environ. Prot..

[42] Kuang C., Xu Y., Lai W., Xie G., Pan Z., Zheng L., Talawar M.P., Ling J., Ye S., Zhou X. (2019). Novel Electrodes for Cathode Electro-Fenton Oxidation Coupled with Anodic Oxidation System for Advanced Treatment of Livestock Wastewater. Electrochim. Acta.

[43] Chadwick D.R., Williams J.R., Lu Y., Ma L., Bai Z., Hou Y., Chen X., Misselbrook T.H. (2020). Strategies to Reduce Nutrient Pollution from Manure Management in China. Front. Agric. Sci. Eng..

[44] Noguera-Méndez P., Molera L., Semitiel-García M. (2016). The Role of Social Learning in Fostering Farmers’ pro-Environmental Values and Intentions. J. Rural Stud..

[45] Wang M.-Y., Lin S.-M. (2020). Intervention Strategies on the Wastewater Treatment Behavior of Swine Farmers: An Extended Model of the Theory of Planned Behavior. Sustainability.

[46] Yang C., Wang J. (2019). Evaluation of Policies on Inappropriate Treatment of Dead Hogs from the Perspective of Loss Aversion. Int J. Environ. Res. Public Health.

[47] Marshall G.R. (2004). From Words to Deeds: Enforcing Farmers’ Conservation Cost-Sharing Commitments. J. Rural Stud..

[48] Boz I. (2018). Determinants of Farmers’ Enrollment in Voluntary Environmental Programs: Evidence from the Eregli Reed Bed Area of Turkey. Environ. Dev. Sustain..

[49] Broens E.M., Graat E.A.M., Van Der Wolf P.J., Van De Giessen A.W., De Jong M.C.M. (2011). Prevalence and Risk Factor Analysis of Livestock Associated MRSA-Positive Pig Herds in The Netherlands. Prev. Vet. Med..

[50] Adams F., Ohene-Yankyera K., Aidoo R., Wongnaa C.A. (2021). Economic Benefits of Livestock Management in Ghana. Agric. Econ..

[51] Schukat S., Heise H. (2021). Smart Products in Livestock Farming—An Empirical Study on the Attitudes of German Farmers. Animals.

[52] Chen G., Tsurumi H. (2010). Probit and Logit Model Selection. Commun. Stat. Theory Methods.

[53] Allison P.D. (1999). Comparing Logit and Probit Coefficients Across Groups. Sociol. Methods Res..

[54] Chen K., Ali M., Veeman M., Unterschultz J., Le T. (2002). Relative Importance Rankings for Pork Attributes by Asian-Origin Consumers in California: Applying an Ordered Probit Model to a Choice-Based Sample. J. Agric. Appl. Econ..

[55] Liljenstolpe C. (2008). Evaluating Animal Welfare with Choice Experiments: An Application to Swedish Pig Production. Agribusiness.

[56] Chen Y., Culpepper S.A. (2020). A Multivariate Probit Model for Learning Trajectories: A Fine-Grained Evaluation of an Educational Intervention. Appl. Psychol. Meas..

[57] Varona L., Noguera J.L., Casellas J., de Hijas M.M., Rosas J.P., Ibáñez-Escriche N. (2020). A Cross-Specific Multiplicative Binomial Recursive Model for the Analysis of Perinatal Mortality in a Diallel Cross among Three Varieties of Iberian Pig. Sci Rep..

[58] Pessoa J., Rodrigues da Costa M., García Manzanilla E., Norton T., McAloon C., Boyle L. (2021). Managing Respiratory Disease in Finisher Pigs: Combining Quantitative Assessments of Clinical Signs and the Prevalence of Lung Lesions at Slaughter. Prev. Vet. Med..

[59] Lu X., White H. (2014). Robustness Checks and Robustness Tests in Applied Economics. J. Econom..

[60] Bianco A.M., Martínez E. (2009). Robust Testing in the Logistic Regression Model. Comput. Stat. Data Anal..

[61] Fine J.P., Bosch R.J. (2000). Risk Assessment via a Robust Probit Model, with Application to Toxicology. J. Am. Stat. Assoc..

[62] Finlay K., Magnusson L.M. (2009). Implementing Weak-Instrument Robust Tests for a General Class of Instrumental-Variables Models. Stata J..

[63] Cai J., Zhang L., Tang J., Pan D. (2019). Adoption of Multiple Sustainable Manure Treatment Technologies by Pig Farmers in Rural China: A Case Study of Poyang Lake Region. Sustainability.

[64] Zhang Y., Ju G., Zhan J. (2019). Farmers Using Insurance and Cooperatives to Manage Agricultural Risks: A Case Study of the Swine Industry in China. J. Integr. Agric..

[65] Haefele M.A., Loomis J.B. (2001). Improving Statistical Efficiency and Testing Robustness of Conjoint Marginal Valuations. Am. J. Agric. Econ..

[66] Gong K., Johnson S., Hill A., Lê J., McKenny A., O’Kane P., Paroutis S., Smith A. (2021). The Bivariate Probit Model in Strategy and Management Research: Applications and Potential. Research in Times of Crisis.

[67] Kwak D.W., Martin R.S., Wooldridge J.M. (2021). The Robustness of Conditional Logit for Binary Response Panel Data Models with Serial Correlation. J. Econom. Methods.

[68] Shimizu I. (2014). Multistage Sampling. Wiley StatsRef: Statistics Reference Online.

[69] Si R., Yao Y., Zhang X., Lu Q., Aziz N. (2021). Investigating the Links Between Vaccination Against COVID-19 and Public Attitudes Toward Protective Countermeasures: Implications for Public Health. Front. Public Health.

[70] Gillespie J.M., Davis C.G., Rahelizatovo N.C. (2004). Factors Influencing the Adoption of Breeding Technologies in U.S. Hog Production. J. Agric. Appl. Econ..

[71] Hinrichs J., Mußhoff O., Odening M. (2008). Economic Hysteresis in Hog Production. Appl. Econ..

[72] Mittal S., Mehar M. (2016). Socio-Economic Factors Affecting Adoption of Modern Information and Communication Technology by Farmers in India: Analysis Using Multivariate Probit Model. J. Agric. Educ. Ext..

[73] Westreich D., Greenland S. (2013). The Table 2 Fallacy: Presenting and Interpreting Confounder and Modifier Coefficients. Am. J. Epidemiol..

[74] Keele L., Stevenson R.T., Elwert F. (2020). The Causal Interpretation of Estimated Associations in Regression Models. Political Sci. Res. Methods.

[75] Dohoo I.R., Ducrot C., Fourichon C., Donald A., Hurnik D. (1997). An Overview of Techniques for Dealing with Large Numbers of Independent Variables in Epidemiologic Studies. Prev. Vet. Med..

[76] Oliver D.M., Zheng Y., Naylor L.A., Murtagh M., Waldron S., Peng T. (2020). How Does Smallholder Farming Practice and Environmental Awareness Vary across Village Communities in the Karst Terrain of Southwest China?. Agric. Ecosyst. Environ..

[77] Min S., Bai J., Huang J., Waibel H. (2018). Willingness of Smallholder Rubber Farmers to Participate in Ecosystem Protection: Effects of Household Wealth and Environmental Awareness. For. Policy Econ..

[78] Wang J., Yang C., Ma W., Tang J. (2020). Risk Preference, Trust, and Willingness-to-Accept Subsidies for pro-Environmental Production: An Investigation of Hog Farmers in China. Environ. Econ. Policy Stud..

[79] Keshavarz M., Karami E. (2016). Farmers’ pro-Environmental Behavior under Drought: Application of Protection Motivation Theory. J. Arid Environ..

[80] Zhu H., Yang J., Xiaowei C. (2019). Application of Modified Gompertz Model to Study on Biogas Production from Middle Temperature Co-Digestion of Pig Manure and Dead Pigs. E3S Web Conf..

[81] LiMei L., YaQing H. (2019). Influencing factors and regulatory strategies on large-scale pig farmers’ environmentally friendly behaviors: An exploratory research based on Grounded Theory. J. Ecol. Rural Environ..

[82] Duong M., Peyre M., Rukkwamsuk T. (2017). Qualitative Assessment of Pig Health Risks Related to the Uses of Food Waste for Pig Production in Sub-Urban. of Hanoi Capital, Vietnam.

[83] Hu Y., Cheng H., Tao S. (2017). Environmental and Human Health Challenges of Industrial Livestock and Poultry Farming in China and Their Mitigation. Environ. Int..

[84] Yu C., Xia B., Qiu M., Du L., Zhang Z., Song X., Xiong X., Hu C., Yang L., Yang C. (2021). Harmless Treatment Method of Dead Chickens in Scale Chicken Farm. E3S Web Conf..

[85] Mao H., Fu Y., Cao G., Chen S. (2021). Contract Farming, Social Trust, and Cleaner Production Behavior: Field Evidence from Broiler Farmers in China. Environ. Sci Pollut Res..

[86] Defrancesco E., Gatto P., Runge F., Trestini S. (2008). Factors Affecting Farmers’ Participation in Agri-Environmental Measures: A Northern Italian Perspective. J. Agric. Econ..

[87] Despotović J., Rodić V., Caracciolo F. (2021). Farmers’ Environmental Awareness: Construct Development, Measurement, and Use. J. Clean. Prod..

[88] Liu N.L.B.H., Kaler J., Ferguson E., O’Kane H., Green L.E. (2018). Sheep Farmers’ Attitudes to Farm Inspections and the Role of Sanctions and Rewards as Motivation to Reduce the Prevalence of Lameness. Anim. Welf. J..

[89] Lu H., Hu L., Zheng W., Yao S., Qian L. (2020). Impact of Household Land Endowment and Environmental Cognition on the Willingness to Implement Straw Incorporation in China. J. Clean. Prod..

[90] Wąs A., Malak-Rawlikowska A., Zavalloni M., Viaggi D., Kobus P., Sulewski P. (2021). In Search of Factors Determining the Participation of Farmers in Agri-Environmental Schemes—Does Only Money Matter in Poland?. Land Use Policy.

